# Metabolic Demands, Center of Mass Movement and Fractional Utilization of $\dot{\text{V}}{\text{O}}_{2\text{max}}$ in Elite Adolescent Tennis Players During On-Court Drills

**DOI:** 10.3389/fspor.2020.00092

**Published:** 2020-07-17

**Authors:** Glenn Björklund, Mikael Swarén, Magnus Norman, Juan Alonso, Fredrik Johansson

**Affiliations:** ^1^Department of Elite Sports Support, The Swedish Sports Confederation, Stockholm, Sweden; ^2^Department of Health Sciences, Swedish Winter Sports Research Centre, Mid Sweden University, Östersund, Sweden; ^3^Swedish Unit of Metrology in Sports, Department of Sports, Fitness and Medicine, Dalarna University, Falun, Sweden; ^4^Swedish Olympic Academy, Stockholm, Sweden; ^5^Good to Great Tennis Academy, Danderyd, Sweden; ^6^Department of Health Promotion Science, Sophiahemmet University, Stockholm, Sweden; ^7^Scandinavian College of Naprapathic Medicine, Stockholm, Sweden

**Keywords:** biomechanics, exercise intensity, motion capture, racket sport, work economy

## Abstract

The aim of the study was to investigate the exercise intensity and energy expenditure during four types of on-court tennis drills. Five female and five male tennis players participated in the study (age: 17 ± 2 years; $\overset{∙}{V}O_{2max}$: 54 ± 6 ml·kg^−1^·min^−1^). Anthropometric measures were taken for each player and, on separate days, each player performed (i) treadmill running to determine $\overset{∙}{V}O_{2max}$ and (ii) four different tennis drills (Drill1-4) during which $\overset{∙}{V}O_{2}$, blood lactate concentration, ratings of perceived exertion (RPE 6–20), and displacement of center of mass (*m*) using 3D kinematics were recorded. The drills were designed to simulate match play with 90 s of rest between each drill. A repeated two-way ANOVA was used for physiological and biomechanical data and Friedman's test for RPE using < α 0.05. Fractional utilization of $\overset{∙}{V}O_{2max}$ was greatest during Drill1 81.8 ± 7.0% and lowest during Drill4 72.4 ± 5.2% (*p* < 0.001) with no difference between sexes (*p* > 0.05). The highest energy expenditure was during Drill1 and lowest during Drill4 (77 ± 15 and 49 ± 11 kcal, respectively, *p* < 0.05). Energy expenditure per meter for Drill1–Drill4 was subsequently reduced for each drill with 10.5 ± 2.1, 9.9 ± 2.2, 7.6 ± 1.7, and 8.0 ± 1.6 J·kg^−1^·m^−1^ (*p* < 0.01). There were no interaction effects for any of these variables. RPE (6–20) and blood lactate concentration post Drill1–Drill4 were 17.5, 15.5, and 13.0 (overall, legs and arms, *p* < 0.001) and 5.9 ± 2.0, 4.9 ± 1.9, 5.6 ± 2.0, and 5.0 ± 2.2 mmol·l^−1^ (*p* < 0.05). The findings of this study demonstrate that the on-court tennis drills performed here are suitable for high intensity training in junior tennis players. The energy expenditure per minute is comparable to similar sports whereas the energy expenditure per meter is notably greater.

## Introduction

Tennis players need to master the complex techniques and sport-specific movements, for example the service motion and the movement patterns on-court, requiring acceleration and deceleration in combination with change of direction (Kovacs, 2006; Kovacs and Ellenbecker, 2011; Hoppe et al., 2014). In addition, tennis has become a true physical challenge requiring strength, speed, power, agility, mobility, aerobic fitness, and anaerobic power output (Baiget et al., 2015). It has been reported that maximal oxygen uptake ($\overset{∙}{V}O_{2max}$) of high-level tennis players is in the range of 44–69 ml·kg^−1^·min^−1^, and above 50 ml·kg^−1^·min^−1^ in the majority of cases (Kovacs, 2006). The average fractional of utilization of $\overset{∙}{V}O_{2max}$ during intensive rallies corresponds to ~50–60% of time spent with $\overset{∙}{V}O_{2max}$ values above 80% (Fernandez et al., 2006). Armstrong and Welsman (1994) report that female athletes generally have 10% lower $\overset{∙}{V}O_{2max}$ than males in childhood and at 16 years of age; the difference often increases to ~35% between sexes. Relative intensity of simulated tennis play and the responses to different training drills showed that drills were stroke-time-dependent, were an increase in number of strokes per drill required significantly greater heart rate, blood lactate concentrations, and oxygen uptake ($\overset{∙}{V}O_{2}$) up to two–three times of $\overset{∙}{V}O_{2max}\;$(Botton et al., 2011; Gomes et al., 2016). In addition, intensity distribution and its relation to aerobic fitness in competitive players appears to determine the intensity players can sustain throughout a game (Baiget et al., 2015). To summarize, energy expenditure expressed as $\overset{∙}{V}O_{2}$ and heart rate responses to standardized tennis drills exhibit large various across different drills (Bekraoui et al., 2012).

In tennis, Global Positioning Systems (GPS), Local Positioning Systems (LPS), and video based systems have been used to track players' movement patterns on-court (Reid et al., 2008, 2016; Hoppe et al., 2014; Whiteside and Reid, 2017). These systems have provided valuable information with sufficient accuracy regarding distances traveled and running patterns but cannot always accurately detect short sprints, high intensity actions, and rapid directional changes, which often occurs in tennis (Barris and Button, 2008; Coutts and Duffield, 2010; Waldron et al., 2011; Sathyan et al., 2012; Vickery et al., 2014; Luteberget et al., 2018). Generally, when compared with females male elite players cover a greater distance per match and at higher speeds (Reid et al., 2016; Whiteside and Reid, 2017). Previous studies have reported the total distance covered per match to be between 2,100–3,200 and 1,200–1,400 m for men and women, respectively (Reid et al., 2016; Pereira et al., 2017; Whiteside and Reid, 2017). However, the difference in the total covered distance per match can be explained by the variable formats of five sets for the men verses three sets for the women as the normalized distance per game or set shows now significant difference between sexes.

Both metabolic monitoring and positioning tracking of the players have been used to quantify tennis players exercise intensity and movements on-court. However, to date these methods have not been used simultaneously in tennis to calculate energy expenditure per meter traveled. As previously reported, both running speed as well as the frequency of turning, affect the energy expenditure during change of direction while running (Hatamoto et al., 2014). Due to the nature of tennis, where there is a constant change of direction, the energy expenditure per meter should be exceptionally high compared to linear sports. Therefore, the aims were to combine physiological and biomechanical measurements to quantify the (i) the fractional utilization of $\overset{∙}{V}O_{2max}$ and (ii) quantify the overall energy expenditure and per meter during four different on-court tennis drills.

## Methods

### Subjects

Ten adolescent elite tennis players from the Swedish national teams, all competing on the International Tennis Federation (ITF) level (five male: age 17 ± 2 years, height 186 ± 7 cm, body mass 73 ± 10 kg; 5 female: age 17 ± 2 years, height 172 ± 3 cm, body mass 69 ± 7 kg), participated in the study. Exclusion criteria were a history of any injuries or illness symptoms during the last 3 months that resulted in rest from training and/or competition for more than 1 week, and/or sickness for more than 3 days during the last 4 weeks before the aerobic and anaerobic tests. All players were right-handed and used two-handed backhand. The calendar year for the players consisted of 20–22 weeks of training with a training volume ranging between 12–20 h per week depending on age; in line with the national recommendations by the Swedish Tennis Association for adolescent elite players. In addition, each player competed for 100–120 matches nationally and/or internationally (ITF) distributed over 22–26 weeks of competition over a calendar year. Therefore, total training and match volume for these players per year accumulates to a range of 500–850 h depending on age and individual schedule. Before any participation, the procedures, and potential risks were explained fully to the subjects. Written informed consent was obtained from each player. The study was in accordance with the Declaration of Helsinki and preapproved by the Regional Ethical Review Board, Stockholm, Sweden (approval no. 2012/1731/2).

### Laboratory Tests

The laboratory tests- were initiated with a progressive 10-min warm-up on a treadmill (Katana, Lode, Groningen, the Netherlands) followed by a 3-min rest period.

The first 5-min of the warm-up consisted of a pre-selected speed of 10 km·h^−1^ for all individuals and thereafter adjusted for each player, according to previous self-reported test results at 3,000 m. The running speed was constant throughout the test while the workload increased by inclination in 1-min steps, 2% at the 1-min mark, and subsequently by 1% until the athlete experienced voluntary exhaustion. Capillary blood samples were collected 1 and 3 min after the cessation of the test. Expired gas and ventilation were measured continuously in mixing chamber mode with a metabolic cart (Jaeger Oxycon Pro, Wuerzburg, Germany). Prior to the start of each test, the gas analyser's turbine were calibrated. If two out of the following three criteria had been met $\overset{∙}{V}O_{2max}$ was considered to be reached: (1) $\overset{∙}{V}O_{2}$ showed a leveling off, defined as an increase in $\overset{∙}{V}O_{2}$ of <150 ml·min^−1^, (2) respiratory exchange ratio exceeded 1.10, and (3) maximal blood lactate samples > 8 mmol·l^−1^. The highest $\overset{∙}{V}O_{2}$ averaged over a period of 60 s was used to calculate $\overset{∙}{V}O_{2max}$.

### Field Tests

#### Tennis Drills

The players performed a 15 min warm up on a bike ergometer (LT2, Monark Exercise AB, Vansbro, Sweden) at 2 W per kilo body mass for the men and 1.5 W per kilo body mass for the women. Following the cycling warm up, the players performed 5 min of individual dynamic movements and stretching, followed by 10 min of hitting warm up. The participants performed three different tennis drills (Drill1-3), divided into 3–6 strokes per set and eight sets per drill and one combination drill (Drill4) of the three previous drills, divided into 3–6 strokes per set and six sets, in total four drills, 30 sets and 130 strokes divided by 90 forehands (69%) and 40 backhands (31%) (Table 1). An experienced professional coach, standing in the center of the court following the player, hand-fed new tennis balls to the player at a speed determined by the completion of the previous shot and movement of the player to the next shot (i.e., self-selected; Reid et al., 2008; Fernandez-Fernandez et al., 2017). Ball placement followed a fixed order presented to the players in advance at a frequency of ~1 ball every 3 s. Each drill was based on movement patters seen in match play and designed by one fitness coach and one ATP coach in agreement. All four drills were carried out consecutively. The start of each set was called out by a research assistant responsible for the timing with a resting time of 25 s between each set and 90 s between each drill. The movement patterns of the three different drills were; Drill1; Spanish Cross) a cross pattern consisting of six strokes, starting position in the center of the court, ball placement starting with a defensive ball on the forehand side, offensive ball on the forehand side, defensive ball on the backhand side, offensive ball on the backhand side, defensive ball on the forehand side, and ending up with an offensive ball on the forehand side, recovery toward the middle of the court after the last shot was instructed to mimic match play. Drill2; Lateral) A lateral pattern consisting of four strokes alternating wide neutral balls to the forehand and backhand side respectively, starting position in the center of the court, starting with a wide ball to the forehand side, ending with a wide ball to the backhand side, recovery toward the middle of the court after the last shot was instructed to mimic match play. Drill3; Inside Out) Inside out forehand, starting position just left of the center of the court, the movement was repeated three times, and recovery toward the middle of the court in between every shot and after the last shot was instructed to mimic match play. Drill 4; Match Simulation) A combination of drill 1, 2, and 3, performed separately and consecutively with 25 s of rest between each set. This was repeated twice. All players were encouraged to move as fast as possible in all four drills and perform strokes with maximal effort, emphasizing the importance to hit the ball inside the court.

**Table 1:** Overview of the drills characteristics.

**Drill**	**Number of sets per drill**	**Rest between each set (s)**	**Rest between each drill (s)**	**Strokes per set**	**Forehand strokes per set**	**Backhand strokes per set**	**Total number of strokes per drill**
1	8	25	90	6	4	2	48
2	8	25	90	4	2	2	32
3	8	25	90	3	3	0	24
4*	2	25	90	6,4,3	4,2,3	2,2,0	26

* *Combination drill consisting of drill 1,2, and 3, repeated two times*.

*m, meters; s, seconds*.

### Oxygen Uptake

All tennis players were equipped with a portable breath-by breath gas analyser (MetaMax3B_R2; Cortex Biophysik GmbH, Leipzig, Germany). The gas analysers were calibrated between the tests for all player using a two-point calibration for the oxygen (*O*_2_) and carbon dioxide (*CO*_2_) sensors. The two-point calibration for the gas sensors used ambient conditions and a mixture of 15% *O*_2_ and 5% *CO*_2_ (UN 1950 Aerosols, Cortex Biophysik GmbH, Leipzig, Germany). The turbine flow was pre checked with a 3 L syringe (M9474-C, Medikro Oy, Kuopio, Finland). The gas analyser was firmly attached to the tennis players to minimize any inconvenience or disturbance to the normal playing patterns. In addition, the facemask was placed in a manner to avoid potential impairment of the vision.

The calculation of energy expenditure *E*_*exp*_ (kcal· min^−1^) was determined by using oxygen uptake ($\overset{∙}{V}O_{2}$) and respiratory exchange ratio (RER) accordingly to the Weir Equation (Weir, 1949).

$$\begin{array}{l} E_{\exp}=\left(\left(1.1\cdot RER\right)+3.9\right)\cdot {\overset{∙}{V}O}_{2} \end{array}$$

Total *E*_*exp*_ for each drill was based on the area under the curve for the $\overset{∙}{V}O_{2}$ data. Each single breath was calculated for $\overset{∙}{V}O_{2}$ and RER as previously described and divided by 60 to get *E*_*exp*_ in seconds times the duration of the breath. Thereafter the sum of every breath's kcal was calculated to get the total kcal for each separate drill.

### Blood Lactate

Capillary blood lactate was collected from fingers on the non-dominant hand. A resting blood lactate was collected before Drill1. Thereafter, collection of capillary blood samples was performed after the completion of each drill. The finger was first wiped with antiseptic solution (Klorhexidin, Fresenius Kabi AB, Uppsala) thereafter capillary blood was sampled using a capillary tube (20 μl) that was dissolved in pre-filled Safe-Lock reaction cup. The pre-filled Safe-Lock cups were then placed in an analyser (Biosen C-line, EKF diagnostic GmhB, Magdeburg, Germany) for blood lactate determination.

### Borg Scale

After completion of all four drills, the players rated their subjective exertion using the 6–20 Rating of Perceived Exertion scale (RPE) and were asked to differentiate between overall, legs and arms RPE (Borg, 1990).

### Biomechanical Measurements

All tennis players used their own individual equipment. The male players wore only tight-fitting shorts and the female players, tight fitting shorts, and a sports bra to be able to attach soft reflective markers directly on the skin. Each marker had a diameter of 15 mm and was attached by double-sided tape and additionally fixed with tape around the base to avoid movement of the markers. Markers were placed on the left and right ASIS (Anterior Superior Iliac Spine), the left and right PSIS (Posterior Superior Iliac Spine) and five markers were attached to the racket. The ASIS and PSIS markers were used to define the movement of the pelvis and the markers on the racket were used to detect and define each shot.

**Figure 1:**
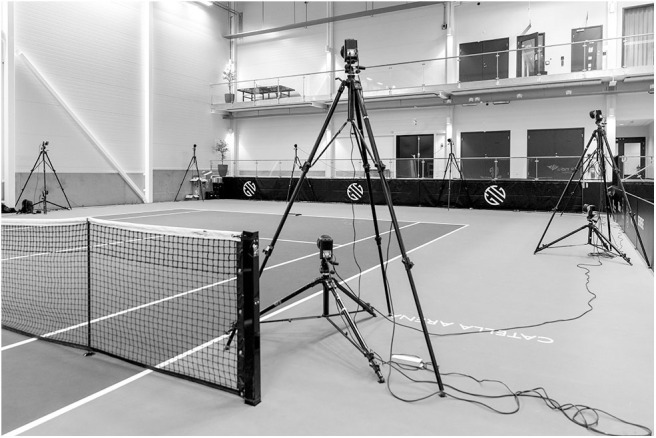
Picture of the calibrated measuring volume with the majority of the motion capture cameras visible.

Kinematic data were collected within a volume 14 × 13 × 5 m by 12 Qualisys Uqus 7+ cameras (Qualisys AB, Gothenburg, Sweden) at 300 Hz and with a resolution of 12 mega pixels, Figure 1. All cameras were places on high tripods around the tennis court, well outside of the sidelines and away from the ball trajectories. The measurement volume was calibrated with a hand-held T-wand, consisting of two reflective markers at each end, with a known distance between them. The orientation of the coordinate system was performed by placing an L-frame at the decided origin which was at the T-point of the baseline. The mean residual for all cameras was 1.4 mm, *SD* = 0.3 mm.

The distance covered by each player per drill, was defined as the horizontal trajectory of the center of mass (COM) in the xy-plane. The position of the center of mass was defined as the center of the pelvis and calculated as the virtual center point of the left and right reflective markers on the ASIS and PSIS. The start of a drill was defined as the first movement of the COM and the end of a drill was defined as the end phase of last shot of the drill. All analyses were performed in Qualisys Track Manager 2019.2 (Qualisys AB, Gothenburg, Sweden), Matlab R2017a (The MathWorks Inc., Natick, MA, USA), and Microsoft Excel (Microsoft Corp. Inc., Redmond, WA, USA).

### Statistical Analysis

All data were checked for normal distribution with Shapiro Wilks test before further analysis. Data were analyzed with SPSS (IBM Corp. Released 2017. IBM SPSS Statistics for Windows, Version 25.0. Armonk, NY: IBM Corp) and jamovi (The jamovi project, 2020). Physiological data were analyzed using a two-way factorial ANOVA with repeated measures. For all ANOVA, Mauchly's sphericity test of the data was checked to control for type one errors and if violated the Green House Geisser correct *F* values were used. If there were global significances for the ANOVA a further Bonferroni *post hoc* analysis was performed. Partial eta squared (${}_{\rho }\eta^{2}$) was used for effect size for the ANOVA. RPE scale outcomes were evaluated with Friedman's test. A Durbin Conover test was applied if the Friedman's test was significant to make pair wise comparisons. All data are presented as mean and ± standard deviation while RPE scale is presented as median and interquartile range (IQR). The α level was set as 0.05 in priori.

## Results

The average covered distances were 31 ± 4, 19 ± 3, 27 ± 2, and 25 ± 6 m, the durations were 14.6 ± 1.4, 9.6 ± 0.9, 10.8 ± 0.5, and 11.3 ± 1.9 s and the mean running speeds were 2.1 ± 0.1, 2.0 ± 0.2, 2.5 ± 0.1, and 2.2 ± 0.1 m·s^−1^ for drill one, two, three, and four, respectively. The percentage spent in different ranges of running speeds for the different drills are presented in Table 2.

**Table 2:** Percentage of time spent in different running speed ranges.

	**Drill1**	**Drill2**	**Drill3**	**Drill4**
**Speed Range**				
0 ≤ 1 m·s^−1^	15 ± 2%	14 ± 4%	11 ± 3%	13 ± 2%
1 ≤ 2 m·s^−1^	27 ± 5%	30 ± 9%	25 ± 3%	26 ± 6%
2 ≤ 3 m·s^−1^	35 ± 3%	42 ± 3%	27 ± 2%	34 ± 5%
3 ≤ 4 m·s^−1^	18 ± 4%	15 ± 7%	27 ± 3%	22 ± 5%
> 4 m·s^−1^	4 ± 2%	1 ± 1%	10 ± 4%	5 ± 4%

### Pre-test Physiological Variables

$\overset{∙}{V}O_{2max}$ for women and men was in absolute values 3.43 ± 0.33 vs. 4.22 ± 0.58 l·min^−1^ (*p* = 0.03, 95% CI−1.5 to −1.0), and in relative values 50 ± 6 vs. 58 ± 3 ml·kg^−1^·min^−1^ (*p* = 0.03, 95% CI−14.0 to −1.1). Maximum HR was for women and men 196 ± 5 vs. 202 ± 4 beats·min^−1^ (*p* > 0.05, 95% CI−12.4 to 0.2) while maximum lactate 12.2 ± 1.6 vs. 12.0 ± 0.7 mmol·l^−1^ (*p* > 0.05, 95% CI−1.6 to 1.9).

### Physiological Variables During On-Court Drills

$\overset{∙}{V}O_{2}$ was highest for the first drill both in absolute values and in relative values (Table 3). The men showed higher absolute $\overset{∙}{V}O_{2}$ values (l·min^−1^) compared to women for all drills, but in relative numbers as fractional utilization (percent of $\overset{∙}{V}O_{2max}$) there were no differences between sexes. The relative $\overset{∙}{V}O_{2}$ (ml·kg^−1^min^−1^) was the greatest at Drill 1 compared to all drills for both women and men while not statistically significant between sexes [*F*_(1, 8)_ = 0.052] the effect size indicated a moderate difference (${}_{\rho }\eta^{2}$ = 0.395). There were no differences in heart rate between drills nor between sexes (Table 3). Resting blood lactate concentration before the start of the drills were 1.5 ± 0.2 and 1.6 ± 0.2 mmol·l^−1^ respectively for women and men (*p* > 0.05). Blood lactate concentration was above 4.0 mmol·l^−1^ throughout all drills but with a variation between drills while no interaction effect for sexes (Table 3). Drill1 had the greatest energy expenditure compared to Drill2 (95% CI = 6.1, 24.1; *p* = 0.002) and Drill4 (95% CI = 19.0, 36.2; *p* < 0.001) but no significant difference compared to Drill3 (CI 95% = −0.7, 23.7; *p* = 0.067. There was no interaction between drill and sex for energy expenditure [*F*_(3, 24)_ = 0.42, *p* < 0.739, ${}_{\rho }\eta^{2}$ = 0.05]. Further no differences in total energy expenditure was observed for sex [*F*_(1, 8)_ = 3.3, *p* = 0.108, ${}_{\rho }\eta^{2}$ = 0.291].

**Table 3:** Physiological variables during the on court tennis drills.

	**Drill1**	**Drill2**	**Drill3**	**Drill4**	***F*-values, *P*-values and effect size**
**Mean VO**_**2**_ **(l·min**^**−1**^**)**					
Women	2.8 ± 0.4^#^^†^	2.7 ± 0.4*^#^	2.6 ± 0.5*^#^	2.6 ± 0.4*^†^	^a^*F*_3, 24_ = 12.6, *p* < 0.001, ${}_{\rho }\eta^{2}$ = 0.612
Men	3.4 ± 0.3^#^^†^	3.2 ± 0.4*	3.3 ± 0.4*	3.0 ± 0.3*^†^	^b^*F*_3, 24_ = 1.3, *p* = 0.291, ${}_{\rho }\eta^{2}$ = 0.141
**Mean VO**_**2**_ **(ml·kg**^**−1**^**·min^−1^)**					
Women	41 ± 4^#^^†^	39 ± 4*	38 ± 5*	37 ± 5*	^a^*F*_3, 24_ = 13.1, *p* < 0.001, ${}_{\rho }\eta^{2}$ = 0.620
Men	47 ± 4^#^^†^	44 ± 3*	45 ± 3*	42 ± 4*	^b^*F*_3, 24_ = 1.3, *p* = 0.296, ${}_{\rho }\eta^{2}$ = 0.140
**Percent of VO**_**2max**_					
Women	82 ± 7^#^^†^	79 ± 7*	77 ± 10*	75 ± 7*	^a^*F*_3, 24_ = 12.7, *p* < 0.001, ${}_{\rho }\eta^{2}$ = 0.613
Men	81 ± 4^#^^†^	75 ± 3*	77 ± 3*	72 ± 5*	^b^*F*_3, 24_ = 1.0, *p* = 0.406, ${}_{\rho }\eta^{2}$ = 0.112
**Percent of HR**_**max**_					
Women	94 ± 4	94 ± 3	95 ± 3	93 ± 3	^a^*F*_3, 24_ = 2.4, *p* = 0.11, ${}_{\rho }\eta^{2}$ = 0.323
Men	91 ± 2	89 ± 4	91 ± 3	91 ± 3	^b^*F*_3, 24_ = 1.4, *p* = 0.278, ${}_{\rho }\eta^{2}$ = 0.220
**Blood Lactate (mmol·l**^**−1**^**)**					
Women	5.9 ± 2.4^†^	5.1 ± 2.0*	6.0 ± 2.2^†^	5.3 ± 2.1	^a^*F*_3, 24_ = 6.1, *p* < 0.01, ${}_{\rho }\eta^{2}$ = 0.441
Men	5.8 ± 1.8^†^	4.8 ± 1.9*	5.3 ± 1.8^†^	4.7 ± 2.4	^b^*F*_3, 24_ = 0.8, *p* = 0.503, ${}_{\rho }\eta^{2}$ = 0.092

*A factorial ANOVA for repeated measurement was used to compare drills and sex with a Bonferroni post-hoc test*.

a *Factorial ANOVA for repeated measurement of drills (4)*.

b *Interaction effect between drills and sex (4 × 2)*.

* *Statistically different from Drill1*.

† *Statistically different from Drill2*.

# *Statistically different from Drill4*.

### Energy Expenditure

**Figure 2:**
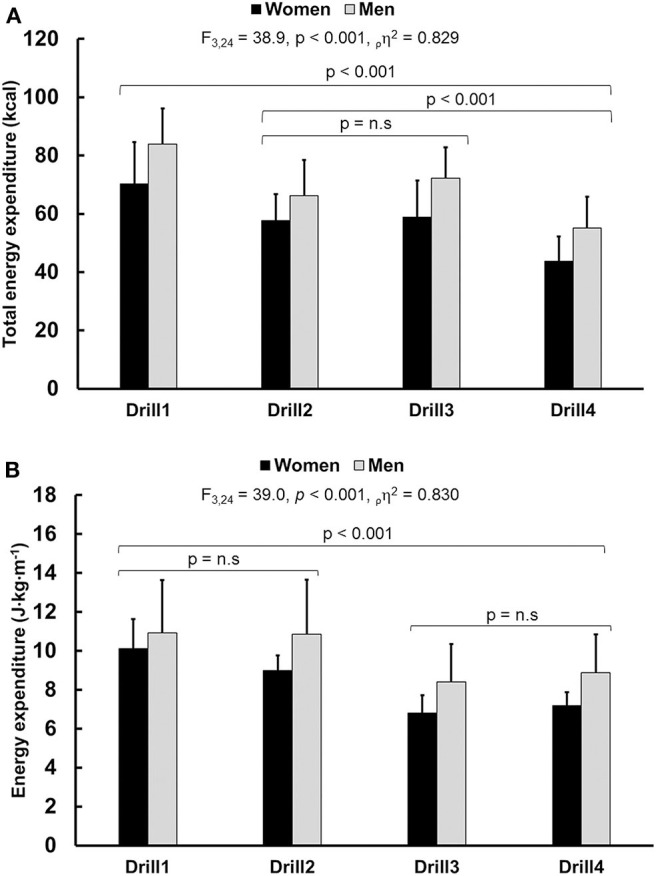
**(A)** Total energy expenditure (kcal) for Drill1-Drill4. **(B)** Energy expenditure per meter for Drill1-4 expressed as joules relative to body weight per meter (J·kg^−1^·m^−1^). Women and men are illustrated using black and gray bars respectively. The *F*-, *P*-values, effect size (${}_{\text{p}}\eta^{2}$) and power values obtained with a two-way ANOVA (Drill × Sex). Non-significant results are presented using n.s. The values given are mean ± *SD*.

The total energy expenditure (kcal) changed between drills 1–4 (Figure 2A). The Drill1 had the greatest energy expenditure compared to Drill2 (95% CI = 6.1, 24.1; *p* = 0.002), Drill4 (95% CI = 19.0, 36.2; *p* < 0.001), and almost to Drill3 (CI 95% = −0.7, 23.7; *p* = 0.067. There was no interaction between drill and sex for energy expenditure [*F*_(3, 24)_ = 0.42, *p* < 0.739, ${}_{\rho }\eta^{2}$ = 0.05] and no differences in total energy expenditure between sexes [*F*_(1, 8)_ = 3.3, *p* = 0.108, ${}_{\rho }\eta^{2}$ = 0.291]. There was a difference in energy expenditure per meter (J·kg^−1^·min^−1^) between drills 1–4 (Figure 2B). The first drill had the greatest energy expenditure compared to Drill3 (95% CI = 1.6, 4.2; *p* < 0.001), Drill4 (95% CI = 1.4, 3.5; *p* < 0.001) while equal energy expenditure to Drill2 (CI 95% = −0.9, 2.1; *p* = 0.199. There was no interaction between drill and sex for energy expenditure [*F*_(3, 24)_ = 1.09, *p* < 0.370, ${}_{\rho }\eta^{2}$ = 0.258] and no differences in total energy expenditure was evident between sexes [*F*_(1, 8)_ = 1.8, *p* = 0.212, ${}_{\rho }\eta^{2}$ = 0.224].

### RPE Scale

**Figure 3:**
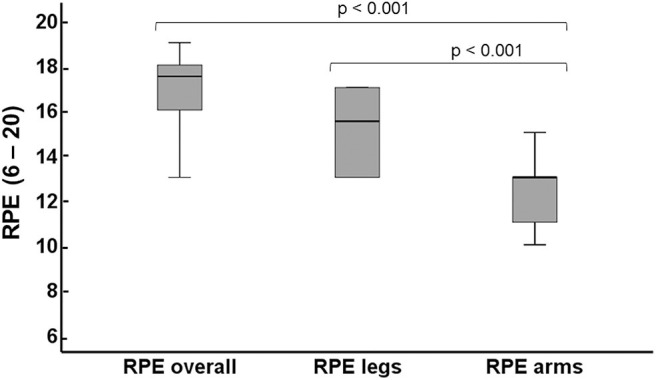
Rate of Perceived Exertion (RPE) for overall, legs and arms. The values given are median and interquartile range (IQR).

RPE differed between overall, leg and arm (χ${}_{2}^{2}$ = 18.0, *p* < 0.001) with RPE overall median of 17.5 (IQR = 16.0 – 18.0), RPE leg median of 15.5 (IQR = 13.5 – 16.8), and RPE arm median 13.0 (IQR = 11.5 – 13.0). The RPE overall was greater than RPE leg and RPE arm (*p* < 0.001 for both RPE leg and arm) with RPE arm being the lowest of all (*p* < 0.001 for both RPE overall and leg) (Figure 3). RPE between women or men did not differ for either overall, leg or arm.

## Discussion

This is the first study that has analyzed the energy expenditure for on-court tennis drills for both total and energy cost per distance. The current study shows that the energy expenditure per distance is exceptionally high for on-court tennis drills and is two to three times greater than linear sports (e.g., running), which substantiate the importance to enhance work economy for tennis players. Additionally, the *O*_2_ demand for the drills used are comparable to previous studies, investigating on-court tennis drills, but in the upper range of previously reported $\overset{∙}{V}O_{2}$ (≥40 ml·kg^−1^·min^−1^). This indicates that the used on-court tennis drills are well-suited for tennis specific high intensity interval training. However, the elevated total energy expenditure per drill suggests that sessions using these drills should be shorter to be able to play with a high technical skill.

### Oxygen Uptake and Energy Expenditure

There are numerous studies that have investigated $\overset{∙}{V}O_{2}$ and distance covered, during both simulated match play and various on-court tennis drills (Smekal et al., 2001; Novas et al., 2003; Reid et al., 2008; Fernandez-Fernandez et al., 2009). However, this is the first study that has combined both methods for calculating energy expenditure per m. While the average $\overset{∙}{V}O_{2}$ during simulated tennis matches is mostly within a range of 50 – 60% of $\overset{∙}{V}O_{2max}$, the present study observed a significantly greater *O*_2_ uptake. The greater average $\overset{∙}{V}O_{2}$ for the drills used in the study is close to 80% of fractional utilization of $\overset{∙}{V}O_{2max}$. However compared to other studies using on-court tennis drills, such high utilization of $\overset{∙}{V}O_{2max}$ are also reported in longer drills used by Gomes et al. (2016). The explanation clearly seems to be associated to both an increase in number of strokes as well as duration of the drills. These longer drills in the study by Gomes et al. (2016) resembles current study in number of strokes per drill, 7–10 vs. 6–8 as well as length, 14–20 vs. 10–15 s. This indicates the impact of number of strokes as well of the duration of the drills on the increase in *O*_2_ uptake. Although the increase in strokes per minute and an elevated *O*_2_ uptake has been reported previously (Cooke and Davey, 2008; Botton et al., 2011) it was suggested that the limb acceleration is a key factor in this association. While running speed is considered to be of less importance for an greater *O*_2_ demand during ground strokes in tennis (Cooke and Davey, 2008), change of direction should not be overlooked. Studies investigating the cost of turning 180 degrees while running showed that change of direction is a major factor that increases the energy expenditure per distance (Hatamoto et al., 2014; Ciprandi et al., 2018). The reason why such a discrepancy could be the result of the use of *O*_2_ per minute and the energy expenditure per distance. Although there are some studies (Botton et al., 2011; Bekraoui et al., 2012) that have used energy expenditure as definition, they have calculated *O*_2_ uptake, which is not equal to energy expenditure expressed as joules or calories (Shaw et al., 2014). Novas et al. (2003) did calculate energy expenditure during tennis play but did not include respiratory exchange ratio and hence excluded variation in substrate utilization (Shaw et al., 2014). Based on the current study, the average *O*_2_ uptake increases with roughly 20–30% while the energy expenditure per distance increases two to three times compared to level running (Fletcher et al., 2009). Interestingly, as shown by Ciprandi et al. (2018), the change of direction resulted in twice as high energy expenditure per distance, this is similar to the results of the present study. Although previous studies have shown that the *O*_2_ uptake increases with strokes per minute (Cooke and Davey, 2008; Botton et al., 2011), the situation with repeated directional changes is also likely to be a major contributor to the increased *O*_2_ uptake in the present study. Nevertheless, the present study shows that the use of on-court drills, $\overset{∙}{V}O_{2}$ is greater than the average fractional utilization of $\overset{∙}{V}O_{2}$ during simulated tennis play. This indicates that these drills would be of great use for tennis specific high intensity training. Moreover, to estimate and understand work economy of on-court tennis play, energy expenditure per distance should be the preferred method over $\overset{∙}{V}O_{2}$. It is also noteworthy that the total energy expenditure is alike in Drill2 and Drill3. However, even though that the mean running speed is higher in Drill3, the energy expenditure per meter is lower in Drill3 as the covered distance in Drill2 is lower. This suggests that the movement pattern is less efficient since players are moving latterly from side to side. Hence, the use of the forehand on the backhand side is more energy demanding which is worth to consider in training as well as during match play to reserve energy, if needed. Similar inference can be made between drill one and four, where drill four consisted of a combination of the other three drills. Hence, the combination of different drills and movement patterns allow the players to move more efficiently at similar speeds.

### Heart Rate and RPE

Heart rate remained at ~90% of max between drills whereas the *O*_2_ cost varied. The inability of heart rate to mirror *O*_2_ uptake is not a new phenomenon during field settings versus laboratory estimations (Crisafulli et al., 2006). More specifically previous studies confirm this finding during tennis play were average heart rate overestimates the energy expenditure compared with $\overset{∙}{V}O_{2}$ measurements with around 20% (Novas et al., 2003). Due to the similar heart rate between drills in current study, heart rate estimations for energy expenditure would have incorrectly been equal in kcal. The most erroneous drill would have been the last one for energy expenditure calculations with the greatest overestimation. However, the use of heart rate seems adequate for internal load, but one should be cautious to make assumptions of precise energy expenditure calculations during tennis play. RPE has been used in many previous studies in tennis (Novas et al., 2003; Mendez-Villanueva et al., 2010). However, the differentiation of RPE for overall, legs and arms has rarely been used as in current study. As shown in current study the legs seem most affected by the drills compared to the arms. This is probably not surprising as and effective movement pattern on-court is decisive for effective tennis play.

### Lactate

While previous studies from simulated tennis matches show rather low blood lactate, concentrations of ~1.4–4.0 mmol·l^−1^ (Fernandez et al., 2006). Such low values are understandably due to the short rallies with a longer rest. Additionally the time spent above the second ventilator threshold during tennis matches is minimal with <5% (Baiget et al., 2015). The current protocols were performed at a clearly elevated blood lactate concentration (~6.0 mmol·l^−1^) compared to tennis match but similar to other on-court drills using similar amount of strokes and rally durations (Gomes et al., 2016). Although the increased lactate response seems to be influenced by the type of drill the most important factor still seems to be rally duration (Reid et al., 2008). Altogether, the drills used in current study clearly stress the anaerobic glycolytic pathways.

### On-Court Distance

Previous studies report the average on-court distance to be 2,000–3,200 and 1,200–1,400 m per match, for men and women respectively (Reid et al., 2016; Cui et al., 2017; Pereira et al., 2017; Whiteside and Reid, 2017). The average distance per rally is reported to be 5–11 m with a duration of 5–12 s, which is shorter compared to the drills in the present study (Murias et al., 2007; Reid et al., 2016; Fenter et al., 2017; Pereira et al., 2017). The majority of running in tennis is performed at speeds of 1–4 m·s^−1^, for both men and women with shorter periods of faster sprints above 3.5 m·s^−1^, which is similar to the four drills in the present study (Martínez-Gallego et al., 2013; Reid et al., 2016; Pereira et al., 2017; Whiteside and Reid, 2017). However, these were obtained from adult professional tennis players and it is plausible that the data are different for adolescent players, as in our study. Based on 10 Hz GPS data, Hoppe et al. (2014) report a total running distance of 3,362 ± 869 m, with the most occurring running speed of 1–2 m·s^−1^ for adolescent tennis players during simulated matches. They do not present any data for running speeds or distances per rally or game. Still, this running speed is lower compared to the total average speed in the drills in the current study (2–2.5 m·s^−1^) which might be because Hoppe et al. (2014) included the walking between each rally whereas our results only include the actual rallies. Another feasible explanation is the use of 10 Hz GPS data, as the accuracy of such a system in tennis is debatable as these systems have been shown to only provide acceptable validity and repeatability for straight running and other team sport specific movements which differ compared to on-court tennis movements (Varley et al., 2012; Galé-Ansodi et al., 2016). Furthermore, Vickery et al. (2014) compared 10 Hz GPS data and 3D motion capture data during tennis play and found that the distance measured using a 10 Hz GPS device differed 13% compared to the 3D motion capture data. Hence, the use of a motion capture system to analyse on-court tennis movements is essential for scientific studies in which the highest accuracy, validity, and repeatability are necessary. The current study, as well as other studies (Vickery et al., 2014; Charbonnier et al., 2015; Fenter et al., 2017) used motion capture systems on-court movements in tennis during field measurements. Hence, in contrary to the statement by Hoppe et al. (2014), motion capture systems can without impracticability, be used for scientific field measurements in tennis. In the current study, the mean residual for all 12 cameras was 1.4 ± 0.3 mm, which vouches for high accuracy and repeatability.

### Limitations

Tennis is an intermittent sport which makes it difficult to use steady state intensities for estimations of energy estimations. However, the use of are under the curve with a breath-by-breath system was the most valid way to quantify the total energy expenditure. The use of Weir equation could underestimate the energy expenditure to a minor degree (Kipp et al., 2018). However, the Weir equation is robust for relative changed between drills. Ciprandi et al. (2018) stressed the importance of alactacid processes and RER into considerations during exercise with change of directions. The present study is the first that considers RER for on-court tennis play. Nevertheless, Ciprandi et al. (2018) suggested that roughly between 0.4 and 1.0 J·kg^−1^·m^−1^ is added by alactacid processes when a maximal velocity close to 75% is used during exercise with change of direction. The high blood lactate concentration in the current study indicate that such numbers likely should be added but the low RER values do not. It can also be argued that that the repeatability of the drills could be increased with the use of a ball machine. However, the players move differently between each stroke, which requires individually timed throws. Still, the standard deviations for the distances and times for all drills indicate a high consistency and repeatability that most likely would have been difficult to surpass with a ball machine.

## Conclusions and Practical Applications

The high energy expenditure during the four different drills indicates that players need to be well prepared to handle the substantial physiological demands of each drill. Furthermore, the overall high demands of $\overset{∙}{V}O_{2max}\;$and anaerobic power presented in our study indicates the importance of high intensity training on- and off-court to improve aerobic fitness and anaerobic power becomes crucial to withstand central and local fatigue. The high energy expenditure is plausibly caused by the multiple changes of direction in combination with a high stroke frequency. Hence, high intensity training on-court to enhance fatigue resistance whilst maintaining stroke mechanics must be emphasized. It is also shown that these high intensity drills do have different energy expenditure that are independent of covered distance or time duration. This knowledge should be considered when planning training sessions and exercises, suggesting that the length of the drills could be modified to further challenge movement skills in combination with technical skills.

## Data Availability Statement

The datasets generated for this study are available on request to the corresponding author.

## Ethics Statement

The studies involving human participants were reviewed and approved by Regional Ethical Review Board, Stockholm, Sweden (approval no. 2012/1731/2). Written informed consent to participate in this study was provided by the participants' legal guardian/next of kin.

## Author Contributions

GB, MS, MN, and FJ designed the study. GB, MS, JA, and FJ performed the experiment, analyzed the data, and prepared the manuscript. All authors read and approved the final manuscript. All authors contributed to the article and approved the submitted version.

## Conflict of Interest

The authors declare that the research was conducted in the absence of any commercial or financial relationships that could be construed as a potential conflict of interest.
